# Alcohol addiction treatments for home resident in Switzerland: review and results of a transectional study

**DOI:** 10.1192/j.eurpsy.2024.815

**Published:** 2024-08-27

**Authors:** I. Gothuey, F. Masdea

**Affiliations:** ^1^ Adult Psychiatric Sector; ^2^Old person psychiatric sector, Firourg Mental Health Network, Marsens, Switzerland

## Abstract

**Introduction:**

In Switzerland, alcohol consumption is even decreasing, with an exception for old people after retirement.35%of them have a heavy or addictive consumption (OFSP, consommation d’alcool en Suisse, fait et chiffres. 01.2023). This is also the case for home residents.

The alcohol consumption by old poeple has negatives consequences on the health (falls, fractures, cognitives disorders).

The autors conduct a crossectionnal enquiry in the homes of Fribourg area to identify addictive behaviour and different existing supports. After a review, the authors present the results of their enquiry

**Objectives:**

Identify how much home-residents have a problematic or an addictive alcohol problem

Identify if there is existing support

Hinghligthing the training need for the staff

**Methods:**

Crossectionnal enquiry was sent in 42 Home of the Fribourg area, with 3 relances

**Results:**

The premliminary result will be completed at the end of 2023:

- Every home identify at leat 4-5 residents with a problematic alcohol consumption
- Most of them, the staff have no specific addictive trainig and no needs for it
- The staff authorise alcohol consumption in the home, to avoid alcohol withdrawal
- The psychiatric consultant in the home can help the staff to manage the counter-attitudes

Home residents are not eligible for specialized addictive care, whil the generally respond well to motivationnal interviewing or to controlled consumption. The lack of staff training could be an hypothesis. The lack of interest in the neagtives conséquences oh alcool on th health of people at the enf of their lives is anothers hypothesis

**Conclusions:**

Nursing home residents are not eligible for specialized addictive care. The enquiry results are astonishing: no need of specialized training, authorization of continous drinking in the different homes, while the literature points the effectivnesse of motivationnal interviewing or controlled approaches by old people with addictive disorders.

Further studies are needed, ethical consideration on the management of alcohol addiction in the elderly should be proposed.

**Disclosure of Interest:**

None Declared

